# Analgesic efficacy and risk of low-to-medium dose intrathecal morphine in patients undergoing cardiac surgery: An updated meta-analysis

**DOI:** 10.3389/fmed.2022.1017676

**Published:** 2022-10-05

**Authors:** I-Wen Chen, Cheuk-Kwan Sun, Ching-Chung Ko, Pei-Han Fu, I-Chia Teng, Wei-Cheng Liu, Chien-Ming Lin, Kuo-Chuan Hung

**Affiliations:** ^1^Department of Anesthesiology, Chi Mei Medical Center, Liouying, Tainan City, Taiwan; ^2^Department of Emergency Medicine, E-Da Hospital, Kaohsiung City, Taiwan; ^3^College of Medicine, I-Shou University, Kaohsiung City, Taiwan; ^4^Department of Medical Imaging, Chi Mei Medical Center, Tainan City, Taiwan; ^5^Department of Health and Nutrition, Chia Nan University of Pharmacy and Science, Tainan City, Taiwan; ^6^Institute of Biomedical Sciences, National Sun Yat-sen University, Kaohsiung City, Taiwan; ^7^Department of Anesthesiology, Chi Mei Medical Center, Tainan City, Taiwan

**Keywords:** analgesia, cardiac surgery, intrathecal morphine, tracheal extubation, respiratory depression

## Abstract

**Background:**

To evaluate the analgesic efficacy and risk of low-to-medium dose intrathecal morphine (ITM) (i.e., ≤0.5 mg) following cardiac surgery.

**Methods:**

Medline, Cochrane Library, Google scholar and EMBASE databases were searched from inception to February 2022. The primary outcome was pain intensity at postoperative 24 h, while the secondary outcomes included intravenous morphine consumption (IMC), extubation time, hospital/intensive care unit (ICU) length of stay (LOS), and ITM-associated side effects (e.g., respiratory depression). Subgroup analysis was performed on ITM dosage (low: <0.3 mg vs. medium: 0.3–0.5 mg).

**Results:**

Fifteen RCTs involving 683 patients published from 1988 to 2021 were included. Pooled results showed significantly lower postoperative 24-h pain scores [mean difference (MD) = −1.61, 95% confidence interval: −1.98 to −1.24, *p* < 0.00001; trial sequential analysis: sufficient evidence; certainty of evidence: moderate] in the ITM group compared to the controls. Similar positive findings were noted at 12 (MD = −2.1) and 48 h (MD = −1.88). Use of ITM was also associated with lower IMC at 24 and 48 h (MD: −13.69 and −14.57 mg, respectively; all *p* < 0.05) and early tracheal extubation (i.e., 48.08 min). No difference was noted in hospital/ICU LOS, and nausea/vomiting in both groups, but patients receiving ITM had higher risk of pruritus (relative risk = 2.88, *p* = 0.008). There was no subgroup difference in IMC except a lower pain score with 0.3–0.5 mg than <0.3 mg at postoperative 24 h. Respiratory depression events were not noted in the ITM group.

**Conclusion:**

Our results validated the analgesic efficacy of low-to-medium dose ITM for patients receiving cardiac surgery without increasing the risk of respiratory depression.

## Introduction

Cardiac surgery, which is traditionally performed *via* median sternotomy and involves extensive tissue retraction and dissection, can be associated with severe pain within postoperative 2 days (1, 2). Not only does uncontrolled pain activate the sympathetic nervous system and increase myocardial oxygen demand by triggering tachycardia, increased cardiac contractility, and hypertension (3), but it could also increase the risks of pulmonary infections and other complications through restricting respiratory capacity, hampering breathing mechanism, and impairing clearance of respiratory secretions (4). Notwithstanding the analgesic effectiveness of high-dose opioid, the associations with potential adverse side effects including prolonged mechanical ventilation, postoperative respiratory complications, and lengthened intensive care unit (ICU) stay have precluded its incorporation into the standard care protocol for patients undergoing cardiac surgery (5, 6). To address this issue, previous studies have shown that central neuraxial blocks (i.e., epidural and intrathecal analgesia) combined with general anesthesia (GA) could attenuate the severity of pain and adrenergic stress response as well as analgesic consumption more effectively compared to parenteral analgesia (7, 8). Indeed, intrathecal analgesia has been gaining popularity for pain control among patients receiving cardiac surgery to alleviate stress response and enhance postoperative recovery (9–11).

Intrathecal morphine (ITM), which enables rapid action of morphine on the central nervous system by enhancing its access to the cerebrospinal fluid, is being increasingly used in a variety of surgeries to provide effective analgesia and decrease opioid consumption (12–15). In addition to its analgesic advantages, other beneficial effects may include a potentially reduced hospital length of stay (LOS) and enhanced recovery after surgery (15–17). However, its use may be associated with side effects such as nausea, vomiting, itching, and even respiratory depression (12–15). Although ITM has been used for decades in patients undergoing cardiac surgery, its possible association with respiratory depression as reported in a previous meta-analysis (i.e., odds ratio of 7.86) has raised a clinical concern that may restrict its application in this patient population (18). On the other hand, pooled evidence has revealed that ITM-associated adverse events are dose-dependent (15, 19). In that meta-analysis involving patients undergoing cardiac and non-cardiac surgeries, a relatively high dosage of ITM (e.g., >0.5 mg or >7 μg/kg) was adopted in over 40% of the included studies (i.e., 11 out of 27 trials) (18). In contrast, focusing on patients undergoing abdominal surgeries, a meta-analysis suggested that ITM with a dose less than 0.5 mg would not increase the risk of respiratory depression (15).

Although ITM has been reported to be a promising analgesic approach for non-cardiac surgery (15), the analgesic efficacy and associated risks of a relatively low-dose ITM remain unclear in those receiving cardiac procedures. As previous meta-analyses have reported an association of an ITM dose of less than 0.5 mg with a prompt extubation without increasing the risk of respiratory depression (8, 14), we investigated the analgesic efficacy and safety of ITM dosage of ≤0.5 mg or ≤7 μg/kg (i.e., based on a total dose ≤0.5 mg for an average adult with a body weight of 70 kg). In the current meta-analysis, a low-dose ITM was defined as that of <0.3 mg as previously reported (20), while we defined a medium-dose ITM as 0.3–0.5 mg. By hypothesizing that low-to-medium dose ITM (i.e., ≤0.5 mg) may provide favorable analgesic efficacy without increasing the risk of respiratory depression in patients undergoing cardiac surgery, this updated meta-analysis attempted to provide updated evidence for clinical guidance through reviewing the currently available clinical trials.

## Methods

This meta-analysis was conducted in accordance with the recommendations of the PRISMA statement and registered with the International Prospective Register of Systematic Reviews (CRD42022310647).

### Data sources and searches

We searched the Cochrane Library, Embase, Google scholar, and Medline databases from inception to February 11, 2022 using the following search terms: [“coronary artery bypass surger*” or “cardiopulmonary bypass surger*” or “cardiovascular surger*” or “cardiac surger*” or “CABG” or “off-pump coronary artery surger*” or “coronary artery bypass graft surger*” or “Heart Surger*” or “Cardiac Surgical Procedure*” or “(Aortic or Mitral or Heart Valve Prosthesis Implantation or Aortic Valve or Mitral Valve) adj4 (procedure* or operation* or surger*)”] and [(“Spinal” or “intraspinal” or “intradural” or “lumbar*” or “theca*” or “intrathecal” or “subarachnoid*” or “sub arachnoid*” or “regional”) adj4 (puncture* or inject* or anesth* or anaesth* or needle*)] limited to randomized controlled trials (RCTs). No restriction was placed on gender, language, study location, and sample size during literature search. The search strategies for one of these databases are demonstrated in Supplementary Table 1. Additional records identified by scrutinizing the reference lists of the retrieved studies were also reviewed for eligibility of being included in the current study.

### Inclusion criteria

To identify articles eligible for the present meta-analysis, we adopted the following criteria: (a) Population: adult patients (age ≥18 years) undergoing a variety of cardiac surgeries with or without cardiopulmonary bypass, (b) Intervention: the use of a low-to-medium dose ITM with or without adjuncts (e.g., local anesthetics or short-acting opioids) as the intervention approach., (c) Comparison: ITM was not administered for postoperative pain control, (d) Outcomes: pain score, intravenous morphine consumption, length of hospital/ICU stay, extubation time, and ITM-associated side effects. We only included RCTs for analysis and contacted the authors of the included articles in which necessary information was missing in an attempt to access the original data.

### Exclusion criteria

Exclusion criteria were: (1) studies which adopted a relatively high-dose ITM (i.e., >0.5 mg or 7 μg/kg); (2) those without a control group; (3) those in which information regarding outcomes was unavailable, and (4) RCTs presented only as letters or abstracts, or (5) those published as reviews, case reports, or other forms instead of original research.

### Study selection

Two authors first independently reviewed the titles and abstracts of the retrieved articles for eligibility of being incorporated into the current study. The same two authors then independently scrutinized the full texts of the potentially eligible studies according to the inclusion and exclusion criteria. Discrepancies in opinions about the suitability of inclusion for a particular RCT were settled through consulting a third reviewer.

### Data extraction

The following information was retrieved from each study: first author, year of publication, patient characteristics, sample size, dosage of ITM, type of surgery, extubation time, intravenous morphine consumption, postoperative pain score, ITM-related side effects (e.g., pruritus, respiratory depression, nausea/vomiting), hospital LOS, ICU LOS. Disagreements were solved through discussion with a third author.

### Outcomes and definitions

The primary outcome was the analgesic efficacy of low-to-medium dose ITM as reflected by the postoperative pain score at postoperative 24 h, while the secondary outcomes included intravenous morphine consumption, extubation time, and hospital/ICU LOS as well as the risks of pruritus, respiratory depression, and nausea/vomiting. The definition of respiratory depression was in accordance with that of each study. If one study did not clearly define this event, we regarded postoperative reintubation or the use of non-invasive ventilation as an indicator of respiratory depression. Subgroup analysis based on the dosage of ITM (i.e., <0.3 mg vs. 0.3–0.5 mg) was also performed to assess possible dose-dependent analgesic efficacy and side effects. Regarding the possible influence of other factors on postoperative 24-h pain score, we conducted subgroup analyses focusing on the impacts of three confounders: (1) the type of surgery [e.g., coronary artery bypass graft surgery (CABG), valve surgery, combined procedures], (2) the use of cardiopulmonary bypass (i.e., yes vs. no), and (3) the use of other intrathecal agents (i.e., ITM alone vs. ITM combined with other agents).

### Assessment of risk of bias

Using the Cochrane’s tool (RoB 2), two authors independently assessed the risks of different biases of the included RCTs, namely, allocation, performance, attrition, measurement, and reporting biases as well as the overall bias (21). The risk of bias of each RCT was reported as “low,” “some concern,” or “high.” Disagreement between the two authors was settled through arbitration that involved a third reviewer.

### Data synthesis and analysis

Cochrane Review Manager (RevMan 5.3; Copenhagen: The Nordic Cochrane Centre, The Cochrane Collaboration, 2014) was used for the present meta-analysis. The pooled risk ratios (RRs) and mean difference (MD) with 95% confidence intervals (CIs) were computed for binary and continuous outcomes, respectively. For the current study, visual analog scale (VAS) 0–10 cm or 0–100 mm, numerical rating scale (NRS) 0–10, and visual numeric scale (VNS) 0–10 were converted into VAS 0–10 cm for pain severity comparison (22). Regarding the comparison of opioid dosage across different studies, we converted all opioid dosages to morphine equivalents as previously described (23). We assessed heterogeneity with *I*^2^ statistics and defined substantial heterogeneity as an *I*^2^ over 50%. Assuming the existence of heterogeneity across the included studies, we adopted *a priori* a random-effects model for outcome evaluation (22, 24). The potential publication bias was assessed by visual inspection of a funnel plot on encountering 10 or more trials sharing a particular outcome. For equivocal findings from funnel plots, Egger’s test was conducted to investigate the possibility of bias using Comprehensive Meta-Analysis version 3.3.070 (BioSTAT, United States). Sensitivity analysis was performed with a leave-one-out approach to weigh the potential influence of the data from an individual trial on the overall outcome. The level of significance was set at <0.05 for all outcome analyses.

Robustness of the conclusion and reliability of the pooled evidence were evaluated with trial sequential analysis (TSA) to reduce false-positive or false-negative outcomes from multiple testing and sparse data (25, 26). TSA was conducted with TSA viewer version 0.9.5.10 Beta^1^. We calculated the required information size as well as the trial sequential monitoring boundaries for all outcomes. The variance was obtained from the retrieved data of our included studies.

If the cumulative *Z*-curve crosses the TSA boundary, there is sufficient evidence for the expected intervention effect with no need for support from further studies. In contrast, if the *Z*-curve fails to cross the TSA boundaries or attain the required information size, the level of evidence is inadequate to support a conclusion. Setting a type I error at 5%, a power at 80%, and a relative risk reduction at 20% for dichotomous outcomes, we computed the required information size with two-sided tests (27).

### Certainty assessment

The certainty of the evidence from our primary and secondary outcomes was assigned to four grades (i.e., high, moderate, low, and very low) by two independent authors based on the probability of study limitations, publication bias, effect consistency, imprecision, and indirectness as described in GRADE. In case of disagreements about certainty ratings, consensus was reached through discussion.

## Results

### Study selection and characteristics

**FIGURE 1:**
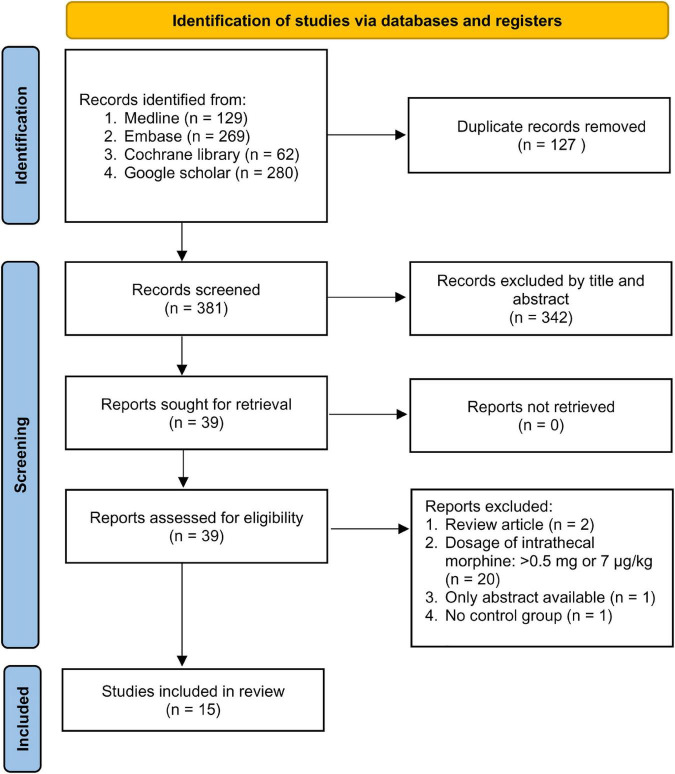
PRISMA flow diagram of study selection for the current meta-analysis.

The study selection process is shown in Figure 1. A total of 740 records were acquired from database search. After removing duplicates and records that did not meet the inclusion criteria, we identified 39 potentially eligible trials for a more detailed review. After analyzing the full text, 24 studies were excluded because of being non-RCTs (review article, *n* = 2), availability only as an abstract (*n* = 1), no control group (*n* = 1), and use of ITM > 0.5 mg or 7 μg/kg (*n* = 20) (Supplementary Table 2). Finally, 15 RCTs published between 1988 and 2021 met our inclusion criteria (3, 28–41). The characteristics of the included trials are shown in Table 1. The mean or median age ranged from 25.9 to 67.3 years with a male predominance (>70%, 11 trials). CABG and mixed CABG/valve surgery were performed in eight (3, 31, 34–36, 39–41) and four (28, 29, 33, 37) trials, respectively, while the other three trials were focused on minimally invasive cardiac surgery (*n* = 2) (30, 38) and valve surgery (*n* = 1) (32). ITM was administered preoperatively in all studies with a maximum dose of 0.5 mg or 7 μg/kg and a minimum dose of 0.25 mg or 0.4 μg/kg. Intrathecal morphine was used as a single agent in 12 trials (3, 28, 30–34, 36, 38–41) and as a component of a combined regimen in three studies (29, 35, 37). Patients in the control groups received local anesthesia of the back, no treatment, or placebo (e.g., intrathecal normal saline). Analysis of the occurrence of respiratory depression including postoperative reintubation or the use of non-invasive ventilation in the five trials with available information (3, 30–33) showed no such incidence in a total of 234 patients, suggesting the safety of its clinical use. Nevertheless, because of the absence of events indicating respiratory depression in all of the five studies, statistical analysis could not be performed.

**TABLE 1:** Characteristics of studies (*n* = 15).

Study	Age (years)^a^	BMI (kg/m^2^) or BW (kg)^a^	*N* ^a^	Male	Procedures	ITM	Time of ITM	Country
Alhashemi (3)	60.4 vs. 64.4	92.6 vs. 90.5	16 vs. 19	34%	CABG	0.25 mg	Preop	Canada
Bettex (28)	53.5 vs. 57.2	76 vs. 81.5	11 vs. 13	92%	CABG/valve surgery	0.5 mg	Preop	Switzerland
Bhat (29)	46 vs. 42	NA	45 vs. 42	43%	CABG/valve surgery	0.25 mg^b^	Preop	India
Dhawan (30)	67.3 vs. 64.5	27.5 vs. 28.6	37 vs. 42	82%	MICS^§^	5 μg/kg	Preop	United States
dos Santos (31)	60.9 vs. 63.8	24.4 vs. 27.1	20 vs. 22	86%	CABG	0.4 mg	Preop	Brazil
Elgendy (32)	26.5 vs. 25.9	56 vs. 64.4	22 vs. 22	48%	AVR	7 μg/kg	Preop	Egypt
Jacobsohn (33)	62 vs. 64	28 vs. 29	22 vs. 21	86%	CABG/valve surgery	6 μg/kg	Preop	United States
Jara (34)	64.4 vs. 64.1	NA	20 vs. 12	78%	CABG^§^	5 μg/kg	Preop	United States
Lena (36)	61 vs. 60	NA	14 vs. 16	77%	CABG	4 μg/kg	Preop	France
Lena (35)	66.4 vs. 66.2	78 vs. 74	20 vs. 20	80%	CABG	4 μg/kg^c^	Preop	France
Lena (37)	66 vs. 66	27 vs. 25	42 vs. 41	80%	CABG/valve surgery	4 μg/kg^d^	Preop	France
Mukherjee (38)	55 vs. 60	25.5 vs. 25.4	30 vs. 31	69%	MICS	1.5 μg/kg	Preop	Germany
Roediger (39)	65.5 vs. 60.7	85 vs. 82.5	15 vs. 15	100%	CABG	0.5 mg	Preop	Belgium
Vanstrum (40)	63.7 vs. 66.8	83.8 vs. 74	16 vs. 14	87%	CABG	0.5 mg	Preop	United States
Yapici (41)	55.3 vs. 59.3	72.8 vs. 62.2	12 vs. 11	70%	CABG	7 μg/kg	Preop	Turkey

AVR, aortic valve replacement; MICS, minimally invasive cardiac surgery; ITM, intrathecal morphine; ^a^present as ITM vs. control group; ^b^combined with 40 mg Marcaine; ^c^combined with clonidine 1 μg/kg; ^d^combined with clonidine 2 μg/kg; Preop, pre-operation; BW, body weight; BMI, body mass index; Coronary artery bypass graft surgery (CABG); ^§^ cardiopulmonary bypass not used.

### Risk of bias assessment

**FIGURE 2:**
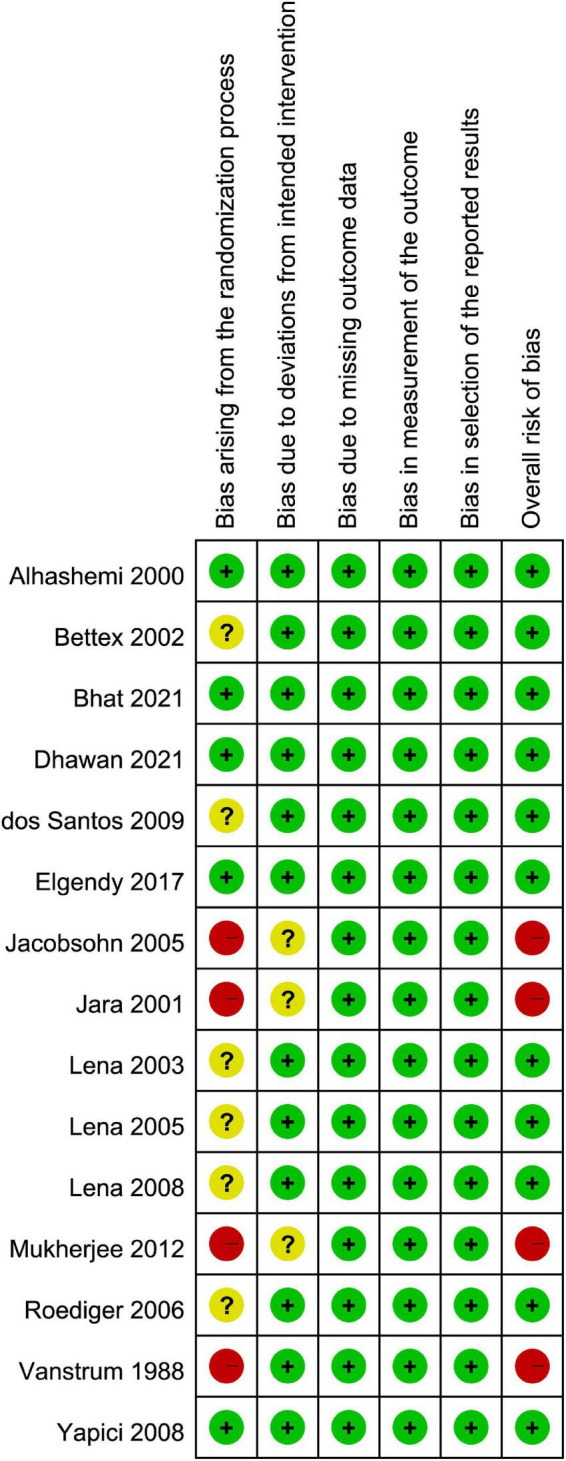
Risks of bias of the included studies.

The assessment of the risk of bias is shown in Figure 2. The overall risk of bias was considered to be low in 11 studies (3, 28–32, 35–37, 39, 41), and high in four trial (33, 34, 38, 40). High risk of bias was associated with bias arising from the randomization process.

### Results of syntheses

#### Primary outcome: Impact of intrathecal morphine on severity of pain at postoperative 24 h

**FIGURE 3:**
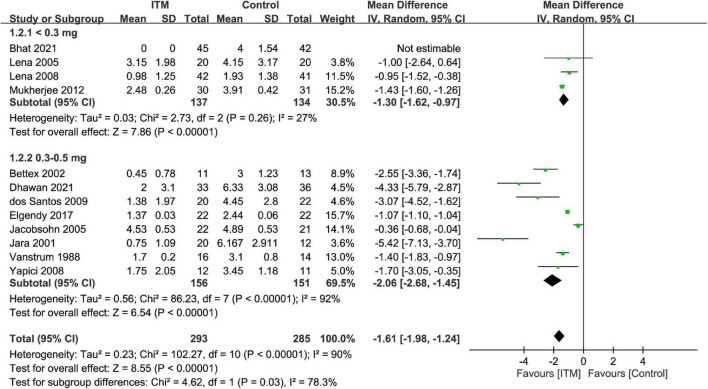
Forest plot comparing the pain score at postoperative 24 h between intrathecal morphine (ITM) and control groups. CI, confidence interval; IV, inverse variance; SD, standard deviation.

By adopting a random-effects model, ITM was associated with a lower pain score compared to that in the control group at postoperative 24 h (MD = −1.61, 95% CI: −1.98 to −1.24, *p* < 0.00001, *I*^2^ = 90%, 11 trials, 578 participants) (Figure 3). There were similar findings at postoperative 12 h (MD = −2.1, 95% CI: −2.83 to −1.36, *p* < 0.00001, *I*^2^ = 96%, 10 trials, 517 participants) and 48 h (MD = −1.88, 95% CI: −2.83 to −0.93, *p* = 0.0001, *I*^2^ = 80%, 4 trials, 259 participants). Subgroup analysis demonstrated a superior analgesic efficacy associated with a dosage of 0.3–0.5 mg compared to that with <0.3 mg (*p* = 0.03) at postoperative 24 h, but not at 12 or 48 h (Supplementary Figures 1, 2).

**FIGURE 4:**
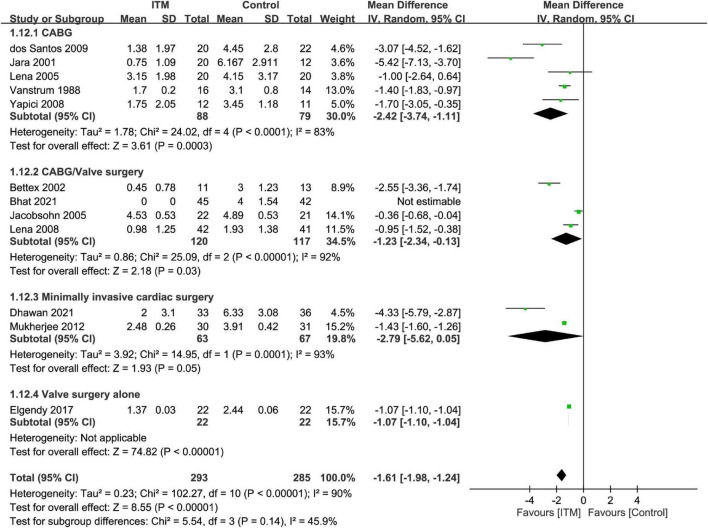
Subgroup analysis comparing postoperative 24-h pain score between intrathecal morphine (ITM) and control groups based on type of cardiac surgery. CI, confidence interval; IV, inverse variance; SD, standard deviation.

**FIGURE 5:**
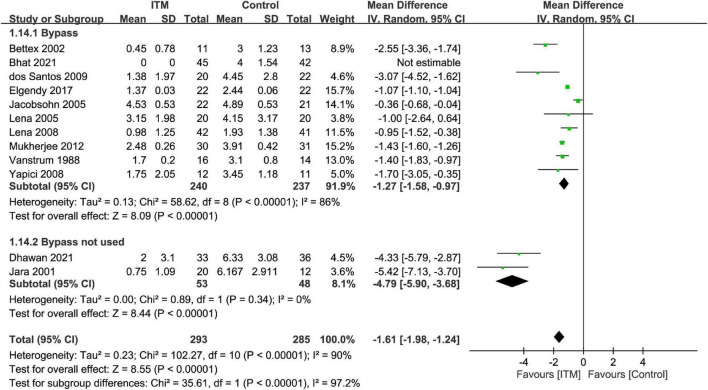
Subgroup analysis comparing postoperative 24-h pain score between intrathecal morphine (ITM) and control groups based on the use of cardiopulmonary bypass. CI, confidence interval; IV, inverse variance; SD, standard deviation.

**FIGURE 6:**
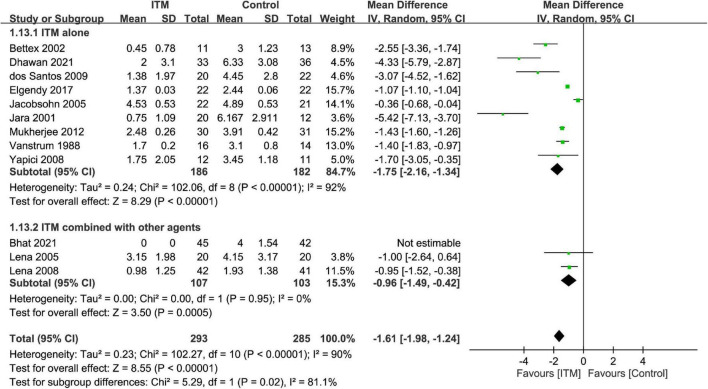
Subgroup analysis comparing postoperative 24-h pain score between intrathecal morphine (ITM) and control groups based on the use of intrathecal agents. CI, confidence interval; IV, inverse variance; SD, standard deviation.

The results of subgroup analyses based on the type of cardiac surgery, the use of cardiopulmonary bypass, and combination with other intrathecal agents are demonstrated in Figures 4–6, respectively. Despite the absence of notable subgroup variation in 24-h pain score among different types of cardiac surgery (*p* = 0.14) (Figure 4), those not subjected to cardiopulmonary bypass (Figure 5) and those receiving ITM alone instead of a combined regimen (Figure 6) were found to have a more significant reduction in 24-h pain score (*p* < 0.00001 and *p* = 0.02, respectively).

#### Secondary outcomes: Association of intrathecal morphine with intravenous morphine consumption, early extubation time, and length of stay

**FIGURE 7:**
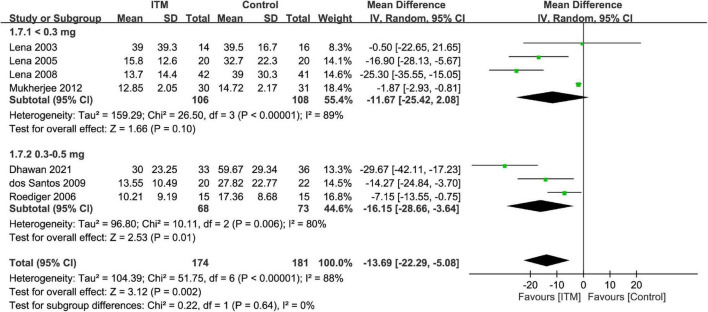
Forest plot comparing intravenous morphine consumption at postoperative 24 h between intrathecal morphine (ITM) and control groups. CI, confidence interval; IV, inverse variance; SD, standard deviation.

Forest plot showed a lower intravenous morphine consumption in the ITM groups than that in the control groups at postoperative 24 h (MD = −13.69, 95% CI: −22.29 to −5.08, *p* = 0.002; I2 = 88%, 355 participants) (Figure 7) and 48 h (MD = −14.57, 95% CI: −26.98 to −2.17, *p* = 0.02; *I*^2^ = 98%, 289 participants) (Supplementary Figure 3). There were no subgroup differences between the doses of 0.3–0.5 mg and <0.3 mg at these two time points.

**FIGURE 8:**
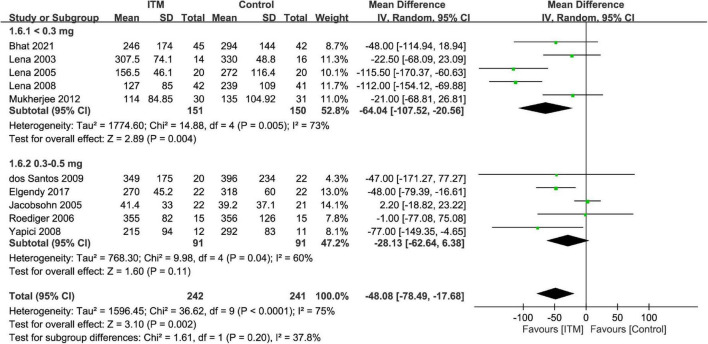
Forest plot comparing extubation time between intrathecal morphine (ITM) and control groups. CI, confidence interval; IV, inverse variance; SD, standard deviation.

The extubation time was 41.4–355 and 39.2–396 min in the ITM and control groups, respectively. Merged results demonstrated a shorter time for tracheal extubation in the ITM group than that in the control group (MD = −48.08 min, 95%: −78.49 to −17.68, *p* = 0.002, *I*^2^ = 75%, 10 trials, 483 participants) (Figure 8). Subgroup analysis revealed no impact of ITM dosage on extubation time (*p* = 0.2).

Our results showed no significant beneficial effect of using ITM on shortening ICU LOS (MD = −5.69 h, 95% CI: −11.83 to 0.46, *p* = 0.07, *I*^2^ = 87%, four trials, 158 participants) (Supplementary Figure 4) or hospital LOS (MD = −0.53 days, 95% CI: −1.16 to 0.1, *p* = 0.1, *I*^2^ = 0, four trials, 178 participants) (Supplementary Figure 5). Subgroup analysis also demonstrated no dose-related impact of ITM on hospital/ICU LOS.

#### Secondary outcomes: Impact of intrathecal morphine on risks of nausea/vomiting and pruritus

Merged results demonstrated no association between ITM and the risk of PONV (RR = 1.13, 95% CI: 0.73 to 1.74, *p* = 0.59, *I*^2^ = 29%, nine trials, 495 participants) (Supplementary Figure 6). Consistently, subgroup analysis showed no impact of ITM on the risk of PONV (*p* = 0.75).

Forest plot revealed a higher risk of pruritus in patients receiving ITM compared to that in the control group (RR = 2.88, 95% CI: 1.31 to 6.31, *p* = 0.008, *I*^2^ = 0%, eight trials, 411 participants) (Supplementary Figure 7). Nevertheless, subgroup analysis demonstrated no correlation between ITM dosage and the risk of pruritus (*p* = 0.92).

### Sensitivity analysis and publication bias

Sensitivity analysis confirmed the robustness of most results except three secondary outcomes (i.e., intravenous morphine consumption at postoperative 24 h, ICU LOS, and risk of pruritus). The potential publication bias was assessed by visual inspection of a funnel plot in three outcomes (i.e., pain score at postoperative 12-, 24 h, and extubation time) (Supplementary Figures 8–10). There is a low risk of publication bias on extubation time (Supplementary Figure 10), while there was uncertainty on pain score at postoperative 12 and 24 h (Supplementary Figures 8, 9). Egger’s test revealed *p*-values of 0.68 and 0.086 for pain score at 12 and 24 h, respectively, indicating no publication bias for the two outcomes.

### Trial sequence analysis

**FIGURE 9:**
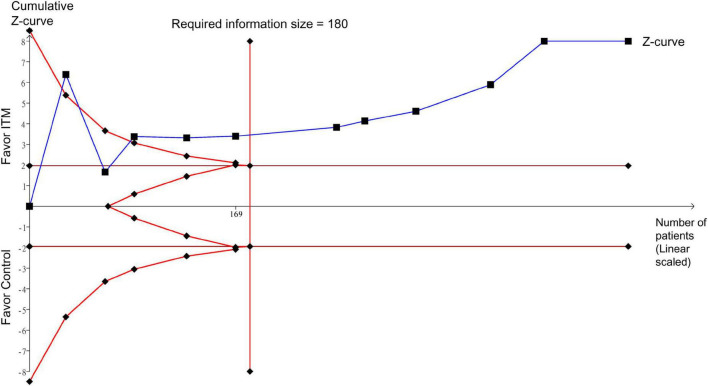
Trial sequence analysis for pain score at postoperative 24 h. ITM, intrathecal morphine.

Trial sequential analysis demonstrated sufficient evidence to support a robust conclusion for pain score at postoperative 24 h (i.e., primary outcome) (Figure 9). In addition, TSA in the current study also suggested a robust conclusion for postoperative pain score (i.e., at 12 and 48 h), intravenous morphine consumption at postoperative 24 h, and extubation time by demonstrating the crossing of cumulative *Z*-curve through the trial sequential monitoring boundary and reaching the required information size (Supplementary Figures 11–13,15). For intravenous morphine consumption at postoperative 48 h, failure of the cumulative *Z*-curve to cross the trial sequential monitoring boundary or reach the required information size on TSA suggested inadequate evidence for this outcome (Supplementary Figure 14). Similarly, the cumulative *Z*-curve did not cross the futility boundary for hospital/ICU LOS and risk of nausea/vomiting, implicating inconclusive evidence for these outcomes (Supplementary Figures 16–18). TSA was not conducted for risk of pruritus due to insufficient information (Supplementary Figure 19).

### Certainty of evidence

Table 2 summarizes the quality of evidence for outcome measures in accordance with the GRADE system. The levels of evidence were graded as low, moderate, and high in two (intravenous morphine consumption at 24 and 48 h), five (pain score at 12–48 h, extubation time, ICU stay), and three (hospital stay, risk of nausea/vomiting, risk of pruritus) outcomes, respectively. The level of evidence was downgraded due to a high degree of inconsistency and imprecision.

**TABLE 2:** Summary of findings for the main comparison.

Outcomes	Effect (Risk or mean)		Relative effect (95% CI)	No. of participants (studies)	Certainty of the evidence (GRADE)	Comments
					
	Intervention group	Control group				
Pain score at 12 h	−	−	MD −2.1 (−2.83 to −1.36)	517 (10 RCTs)	ⴲⴲⴲ◯ Moderate	b
Pain score at 24 h	−	−	MD −1.61 (−1.98 to −1.24)	578 (11 RCTs)	ⴲⴲⴲ◯ Moderate	b
Pain score at 48 h	−	−	MD −1.88 (−2.83 to −0.93)	259 (4 RCTs)	ⴲⴲⴲ◯ Moderate	b
Intravenous morphine consumption at 24 h	−	−	MD −13.69 (−22.29 to −5.08)	355 (7 RCTs)	ⴲⴲ◯◯ Low	a, b
Intravenous morphine consumption at 48 h	−	−	MD −14.57 (−26.98 to −2.17)	289 (5 RCTs)	ⴲⴲ◯◯ Low	a, b
Extubation time	−	−	MD −48.08 (−78.49 to −17.68)	483 (10 RCTs)	ⴲⴲⴲ◯ Moderate	b
Intensive care unit (ICU) length of stay	−	−	MD −5.69 (−11.83 to 0.46)	158 (4 RCTs)	ⴲⴲⴲ◯ Moderate	b
Hospital stays	−	−	MD −0.53 (−1.16 to 0.1)	178 (4 RCTs)	ⴲⴲⴲⴲ High	−
Nausea/vomiting	56/251	48/244	RR 1.13 (0.73 to 1.74)	495 (9 RCTs)	ⴲⴲⴲⴲ High	−
Pruritis	22/209	6/202	RR 2.88 (1.31 to 6.31)	411 (8 RCTs)	ⴲⴲⴲⴲ High	−

^a^Wide 95% CI.

^b^The I square is more than 50%.

GRADE Working Group grades of evidence: High certainty: we are very confident that the true effect lies close to that of the estimate of the effect. Moderate certainty: we are moderately confident in the effect estimate: the true effect is likely to be close to the estimate of the effect, but there is a possibility that it is substantially different. Low certainty: our confidence in the effect estimate is limited: the true effect may be substantially different from the estimate of the effect. Very low certainty: we have very little confidence in the effect estimate: the true effect is likely to be substantially different from the estimate of effect.

## Discussion

Satisfactory postoperative pain control is essential to patient recovery after cardiothoracic surgery because inadequate analgesia may contribute to prolonged immobilization as well as impaired lung expansion and respiratory function, especially in those undergoing median sternotomy (4, 42–44). Our results demonstrated an association of low-to-medium dose ITM with a lower pain score and intravenous morphine consumption compared to the control group up to postoperative 48 h without increasing the risks of PONV and respiratory depression. Besides, a shorter extubation time (i.e., 48.08 min) was noted in patients receiving low-to-medium dose ITM despite the absence of a positive impact of ITM on ICU/hospital LOS. On the other hand, ITM-associated pruritus was noted regardless of the dosage used in the current meta-analysis.

Although two previous meta-analyses recruiting patients receiving cardiac (8) or cardiac/non-cardiac (18) surgery reported the effectiveness of ITM for reducing pain score and intravenous morphine consumption, most trials in one meta-analysis (i.e., 13 out of 17) (8) and a significant proportion in the other (i.e., 11 out of 27) (18) used a relatively high dose of ITM (i.e., 8 μg/kg–4 mg). Therefore, the relatively high risk of respiratory depression (odds ratio: 7.86) in one of the meta-analyses (18), which may partly be attributed to a high ITM dosage, raises the concern over the possibility of a dose-related increase in the risk of respiratory complications. Similarly, despite focusing on patients receiving CABG, another meta-analysis including mostly trials adopting a high-dose ITM (8) could not reflect the efficacy of low-to-medium dose ITM in the cardiac surgery setting. Accordingly, the present study, which systematically reviewed the evidence from currently available clinical trials, is the first to investigate the impacts of low-to-medium dose ITM on the efficacy of postoperative analgesia as well as the risks of adverse side-effects in patients after cardiac surgery.

In general, surgical pain after cardiac procedures is most intense during the first 2 days, especially in the younger population (1). Compared with previous meta-analyses which did not investigate the analgesic efficacy of ITM at postoperative 48 h (8, 18), our finding of a significant reduction in pain intensity associated with low-to-medium dose ITM at postoperative 12–48 h (range of mean difference: −1.61 to −2.1) highlighted its efficacy during the acute painful period. In addition, subgroup analysis indicated no impact of ITM dosage on analgesic efficacy at postoperative 12 and 48 h, implying the feasibility of adopting a low-dose ITM (i.e., <0.3 mg or 4 μg/kg) in the cardiac operation setting.

Despite the lack of a significant beneficial impact of ITM on mortality or the incidence of myocardial infarction following cardiac surgery from pooled evidence (8, 45), optimization of acute pain management with ITM not only may enhance postoperative recovery and minimize the possibility of persistent chronic pain following cardiac surgery (46, 47) but could also reduce the risk of postoperative delirium, which has been identified as a potential sequela of acute pain (48, 49) possibly associated with long-term cognitive decline (50). Hence, our findings suggested that incorporation of ITM into the standard pain management strategy may be recommended for this patient population.

In the present study, ITM was related to a lower intravenous morphine consumption compared with the control groups at postoperative 24 (MD = −13.69 mg) and 48 (MD = −14.57 mg) hours. Consistent with our findings, a previous meta-analysis in which the majority of included trials used a high-dose ITM (i.e., 8 μg/kg–4 mg) reported that ITM decreased intravenous morphine consumption by 11 mg after cardiac surgery (8). The comparable reductions in intravenous morphine dosage between the present study and the previous meta-analysis (8) suggested similar opioid-sparing effects between high-dose (i.e., 8 μg/kg–4 mg) and low-to-medium dose (i.e., ≤0.5 mg) ITM in the cardiac surgery setting. Furthermore, we also found no impact of ITM dosage on intravenous morphine consumption during subgroup analysis (i.e., <0.3 mg vs. 0.3–0.5 mg), implying the feasibility of using a low-dose ITM in clinical practice. Nevertheless, compared with the control group with a median intravenous morphine consumption of 32.7 mg at postoperative 24 h, our study showed a reduction in intravenous morphine dosage only by only 13.69 mg in those receiving low-to-medium dose ITM. Therefore, our findings implied the need for additional postoperative analgesic strategies in patients after cardiac surgery.

Despite the lack of clinical significance, we revealed a shorter extubation time in the ITM group compared to that in the control group (i.e., MD = −48.08 min). This finding may be attributed to a decreased pain intensity and reduced intravenous morphine consumption in the immediate postoperative period (51). In contrast, the use of a relatively high-dose ITM, which could be associated with respiratory depression (15), may mask the beneficial effect of ITM on early tracheal extubation in a previous meta-analysis (8). Taking into account the recommended practice of early extubation (defined as within postoperative 6 h) after cardiac surgery (52) that was demonstrated in our control group, a further reduction of 48.08 min within such a relatively short period by using ITM as shown in the present study could be of clinical significance. Such a tendency for early tracheal extubation in the current meta-analysis may partially explain the relatively minor shortening in extubation time with low-to-medium dose ITM. As early tracheal extubation has been found to be associated with a decreased risk of infections, stroke, renal failure, and mortality (53–55), our results suggested that adoption of low-to-medium dose ITM in patients with a high risk of delayed extubation [e.g., the elderly (56)] may be recommended.

There are several limitations that need to be addressed in the current meta-analysis. First, the relatively small sample size of each trial included in the present study may potentially bias our results. Second, the recruitment of predominantly males (i.e., ≥70% in 11 out of 15 trials) with a relatively young age (i.e., ≤65 years) in our study may restrict the applicability of our findings to females and the aged population. Third, heterogeneity in study design, procedure, drug dosage, and institute-based practices across the included studies may bias our study outcomes. In fact, our finding of a high heterogeneity in pain score and intravenous morphine consumption implicated a potential adverse effect on the reliability of our results. Fourth, the availability of only five trials that provided information about the absence of respiratory depression warrants further investigations into the potential influence. Fifth, because the analgesic efficacy of ITM may be affected by the use of other adjuncts or cardiopulmonary bypass, further studies are needed to address this issue. Finally, the beneficial effects of low-to-medium dose ITM on the risk of mortality and myocardial infarction were not investigated because of limited information available from the included studies.

## Conclusion

Our results demonstrated that low-to-medium dose intrathecal morphine (i.e., ≤0.5 mg) was associated with a lower pain severity and intravenous morphine consumption without increasing the risk of respiratory depression. Nevertheless, our finding of only a moderate reduction in intravenous morphine consumption associated with the use of low-to-medium dose ITM warrants further studies to investigate the effectiveness of a multimodal analgesic approach in the post-cardiac surgery care setting.

## Author contributions

I-WC and C-KS contributed to conceptualization and literature search. C-CK and W-CL contributed to methodology. C-CK and P-HF contributed to trial selection. K-CH contributed to data analysis. P-HF and I-CT contributed to data extraction. K-CH, C-ML, and C-KS contributed to writing—original draft preparation. K-CH and C-KS contributed to writing—review and editing. All authors have read and agreed to the published version of the manuscript.

## Footnotes

1
www.ctu.dk/tsa
