# Supplementary material for: Analgesic efficacy and risk of low-to-medium dose intrathecal morphine in patients undergoing cardiac surgery: An updated meta-analysis
Source: Front Med (Lausanne). 2022 Oct 5;9:1017676. doi: 10.3389/fmed.2022.1017676 (PMC9581243; doi:10.3389/fmed.2022.1017676)

**Appendix**

**Supplemental Table 1.** Search strategies for Medline

**Supplemental Table 2.** Studies excluded and reasons

**Supplemental Figure 1.** Forest plot comparing the pain score at postoperative 12 hours

**Supplemental Figure 2.** Forest plot comparing the pain score at postoperative 48 hours

**Supplemental Figure 3:** Forest plot comparing the morphine consumption at postoperative 48 hours

**Supplemental Figure 4:** Forest plot comparing the length of stay at intensive care unit

**Supplemental Figure 5**: Forest plot comparing the length of hospital stay

**Supplemental Figure 6**: Forest plot comparing the risk of nausea/vomiting

**Supplemental Figure 7**: Forest plot comparing the risk of pruritis

**Supplemental Figure 8**: Funnel plot for pain score at postoperative 12 hours.

**Supplemental Figure 9**: Funnel plot for pain score at postoperative 24 hours.

**Supplemental Figure 10**: Funnel plot for extubation time.

**Supplemental Figure 11**: Trial sequence analysis for pain score at postoperative 12 hours

**Supplemental Figure 12**: Trial sequence analysis for pain score at postoperative 48 hours

**Supplemental Figure 13.** Trial sequence analysis for morphine consumption at postoperative 24 hours

**Supplemental Figure 14.** Trial sequence analysis for morphine consumption at postoperative 48 hours

**Supplemental Figure 15.** Trial sequence analysis for extubation time

**Supplemental Figure 16.** Trial sequence analysis for intensive care unit stay

**Supplemental Figure 17.** Trial sequence analysis for hospital stay

**Supplemental Figure 18.** Trial sequence analysis for postoperative nausea/vomiting

**Supplemental Figure 19.** Trial sequence analysis for postoperative pruritis

**Supplemental Table 1.** Search strategies for Medline

| 1 | ("coronary artery bypass surger*" or "cardiopulmonary bypass surger*" or "cardiovascular surger*" or "cardiac surger* " or "CABG" or "off-pump coronary artery surger*" or "coronary artery bypass graft surger*" or "Heart Surger*" or "Cardiac Surgical Procedure* " or "(Aortic or Mitral or Heart Valve Prosthesis Implantation or Aortic Valve or Mitral Valve) adj4 (procedure* or operation* or surger*)").mp. |
| --- | --- |
| 2 | exp "Cardiac Surgical Procedures"/ |
| 3 | (("Spinal" or "intraspinal" or "intradural" or "lumbar*" or "theca*" or "intrathecal" or "subarachnoid*" or "sub arachnoid*" or "regional") adj4 (puncture* or inject* or anesth* or anaesth* or needle*)).mp. |
| 4 | exp "Anesthesia, Spinal"/ or exp "Injections, Spinal"/ or exp "Spinal Puncture"/ |
| 5 | (1 or 2) and (3 or 4) |
| 6 | 5 and (((randomized controlled trial or controlled clinical trial).pt. or randomi*ed.ab. or placebo.ab. or drug therapy.fs. or randomly.ab. or trial.ab. or groups.ab.) not (exp animals/ not humans.sh.)) |

**Supplemental Table 2.** Studies excluded and reasons

| Reasons for exclusion | Reference |
| --- | --- |
| Dosage of intrathecal morphine 1 mg | [1-3] |
| Dosage of intrathecal morphine 2 mg | [4-6] |
| Dosage of intrathecal morphine 4 mg | [7, 8] |
| Dosage of intrathecal morphine 8 ug/kg | [9-12] |
| Dosage of intrathecal morphine 10 ug/kg | [13-17] |
| Dosage of intrathecal morphine 15 ug/kg | [18] |
| Dosage of intrathecal morphine 20 ug/kg | [19, 20] |
| Only abstract available | [21] |
| No control group available | [22] |

[1] Arya VK, Yaddanapuri L, Madhusudan P, Thingnam SK, Bahl A. High spinal anesthesia in severe mitral stenosis: Comparison of hemodynamics with high dose opioid anesthesia. Anesthesia and Analgesia. 2012;1):S72.

[2] Fitzpatrick GJ, Moriarty DC. Intrathecal morphine in the management of pain following cardiac surgery. A comparison with morphine i.v. British Journal of Anaesthesia. 1988;60:639-44.

[3] Hall R, Adderley N, MacLaren C, McIntyre A, Barker R, Imrie D, et al. Does intrathecal morphine alter the stress response following coronary artery bypass grafting surgery? Canadian Journal of Anaesthesia. 2000;47:463-6.

[4] Bowler I, Djaiani G, Abel R, Pugh S, Dunne J, Hall J. A combination of intrathecal morphine and remifentanil anesthesia for fast-track cardiac anesthesia and surgery. Journal of Cardiothoracic and Vascular Anesthesia. 2002;16(6):709-14.

[5] Lenkutis T, Bieliunas A, Gedminaite I. [Intrathecal morphine for postoperative analgesia in cardiac surgery]. Medicina (Kaunas, Lithuania). 2002;38 Suppl 2:221-3.

[6] Aun C, Thomas D, St John-Jones L, Colvin MP, Savege TM, Lewis CT. Intrathecal morphine in cardiac surgery. Eur J Anaesthesiol. 1985;2:419-26.

[7] Chaney MA, Smith KR, Barclay JC, Slogoff S. Large-dose intrathecal morphine for coronary artery bypass grafting. Anesth Analg. 1996;83:215-22.

[8] Sebel PS, Aun C, Fiolet J, Noonan K, Savege TM, Colvin MP. Endocrinological effects of intrathecal morphine. Eur J Anaesthesiol. 1985;2:291-6.

[9] Latham P, Zarate E, White PF, Bossard R, Shi C, Morse LS, et al. Fast-track cardiac anesthesia: a comparison of remifentanil plus intrathecal morphine with sufentanil in a desflurane-based anesthetic. J Cardiothorac Vasc Anesth. 2000;14:645-51.

[10] Martinez A, Oslaida M, Paneque RJ, Escobar IC, Bouza RC, Pratts AC. Neuroaxial anesthesia methods combined with general anesthesia for beating heart surgery. British Journal of Anaesthesia. 2012;2):ii215.

[11] Mehta Y, Kulkarni V, Juneja R, Sharma KK, Mishra Y, Raizada A, et al. Spinal (subarachnoid) morphine for off-pump coronary artery bypass surgery. Heart Surg Forum. 2004;7:E205-10.

[12] Zarate E, Latham P, White PF, Bossard R, Morse L, Douning LK, et al. Fast-track cardiac anesthesia: use of remifentanil combined with intrathecal morphine as an alternative to sufentanil during desflurane anesthesia. Anesthesia & Analgesia. 2000;91:283-7.

[13] Chaney MA, Furry PA, Fluder EM, Slogoff S. Intrathecal morphine for coronary artery bypass grafting and early extubation. Anesth Analg. 1997;84:241-8.

[14] Chaney MA, Nikolov MP, Blakeman BP, Bakhos M. Intrathecal morphine for coronary artery bypass graft procedure and early extubation revisited. J Cardiothorac Vasc Anesth. 1999;13:574-8.

[15] Shroff A, Rooke GA, Bishop MJ. Effects of intrathecal opioid on extubation time, analgesia, and intensive care unit stay following coronary artery bypass grafting. Journal of Clinical Anesthesia. 1997;9(5):415-9.

[16] Turker G, Goren S, Sahin S, Korfali G, Sayan E. The efficacy and safety of intrathecal morphine combined with remifentanil anesthesia for off-pump coronary artery bypass surgery. [Turkish]. Gogus-Kalp-Damar Anestezi ve Yogun Bakim Dernegi Dergisi. 2004;10(2):67-74.

[17] Turker G, Goren S, Sahin S, Korfali G, Sayan E. Combination of intrathecal morphine and remifentanil infusion for fast-track anesthesia in off-pump coronary artery bypass surgery. J Cardiothorac Vasc Anesth. 2005;19:708-13.

[18] Tamayo E, Soria S, Martinez-Martinez A, Martinez-Escribano A, Rodriguez R, Munoz F, et al. Postoperative analgesia in cardiac surgery: spinal versus intravenous morphine. [Spanish]. Revista espanola de anestesiologia y reanimacion. 2006;53(3):145-51.

[19] Boulanger A, Perreault S, Choinière M, Priéto I, Lavoie C, Laflamme C. Intrathecal morphine after cardiac surgery. Ann Pharmacother. 2002;36:1337-43.

[20] Casey WF, Wynands JE, Ralley FE, Ramsay JG, O'Connor JP, Katz JM, et al. The role of intrathecal morphine in the anesthetic management of patients undergoing coronary artery bypass surgery. J Cardiothorac Anesth. 1987;1:510-6.

[21] Koch E, Scholz M, Mukherjee C, Banusch J, Ender J. Low dose intrathecal morphine does not prolong extubation time after minimally invasive cardiac surgery. Journal of Cardiothoracic and Vascular Anesthesia. 2011;1):S24-S5.

[22] Nader ND, Li CM, Dosluoglu HH, Ignatowski TA, Spengler RN. Adjuvant therapy with intrathecal clonidine improves postoperative pain in patients undergoing coronary artery bypass graft. Clinical Journal of Pain. 2009;25(2):101-6.

**Supplemental Figure 1.** Forest plot comparing the pain score at postoperative 12 hours between intrathecal morphine (ITM) and control groups. CI, confidence interval; IV, inverse variance; SD, standard deviation.


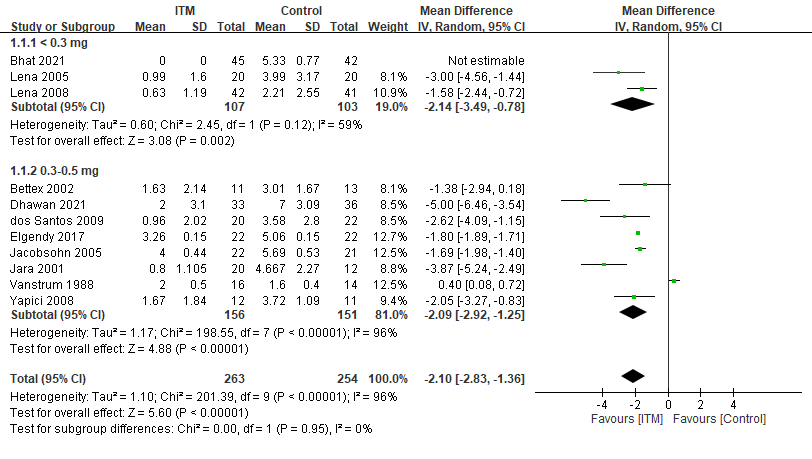


**Supplemental Figure 2.** Forest plot comparing the pain score at postoperative 48 hours between intrathecal morphine (ITM) and control groups. CI, confidence interval; IV, inverse variance; SD, standard deviation.


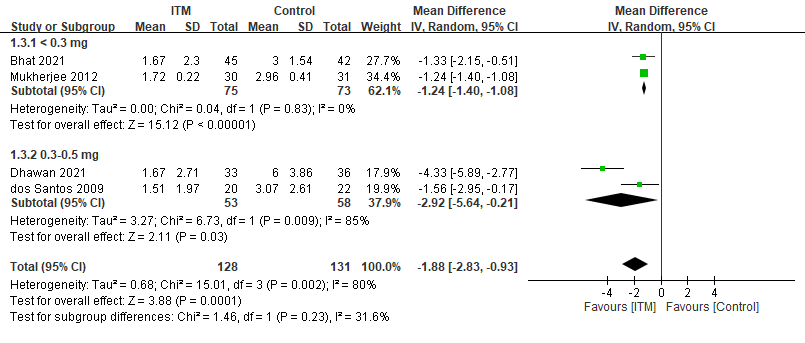


**Supplemental Figure 3:** Forest plot comparing the morphine consumption at postoperative 48 hours between intrathecal morphine (ITM) and control groups. CI, confidence interval; IV, inverse variance; SD, standard deviation.


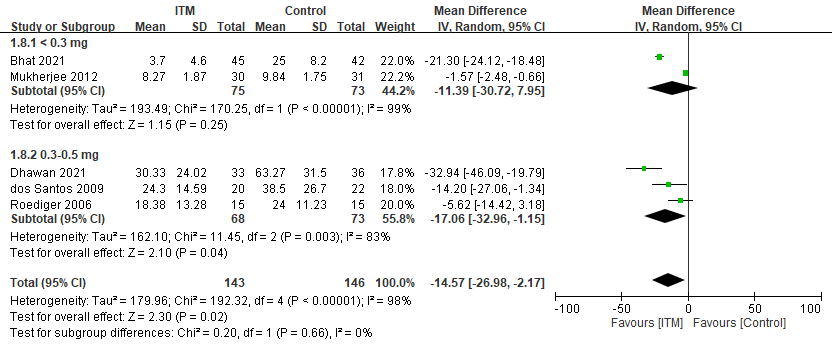


**Supplemental Figure 4:** Forest plot comparing the length of stay at intensive care unit between intrathecal morphine (ITM) and control groups. CI, confidence interval; IV, inverse variance; SD, standard deviation.


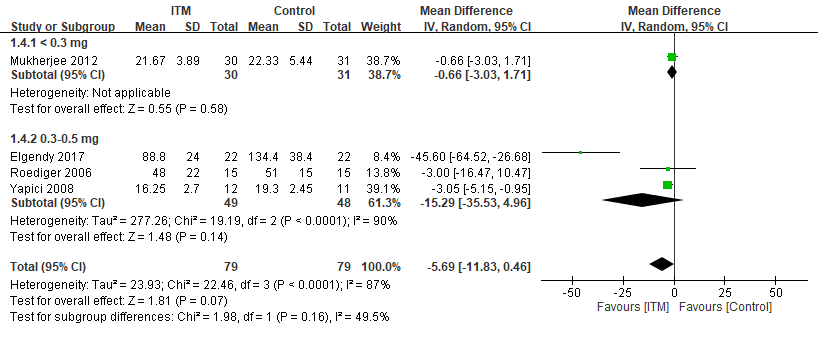


**Supplemental Figure 5**: Forest plot comparing the length of hospital stay between intrathecal morphine (ITM) and control groups. CI, confidence interval; IV, inverse variance; SD, standard deviation.


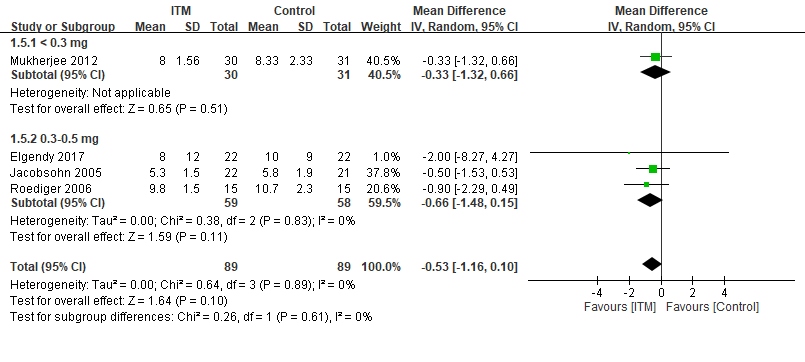


**Supplemental Figure 6**: Forest plot comparing the risk of nausea/vomiting between intrathecal morphine (ITM) and control groups. CI, confidence interval.


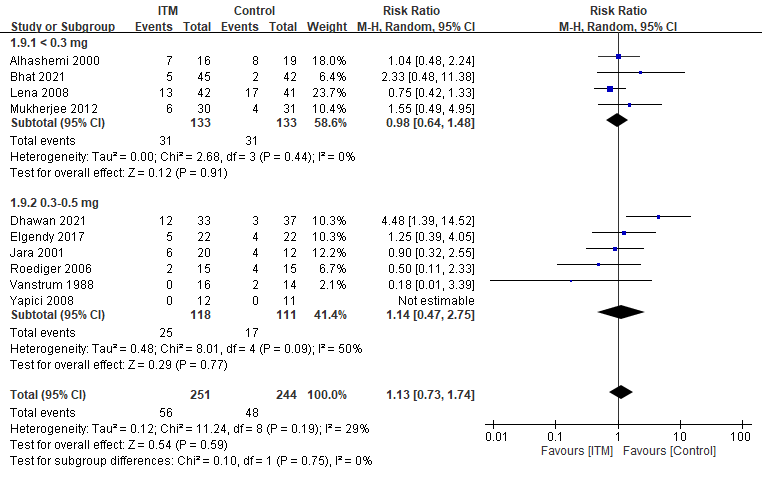


**Supplemental Figure 7**: Forest plot comparing the risk of pruritis between intrathecal morphine (ITM) and control groups. CI, confidence interval.


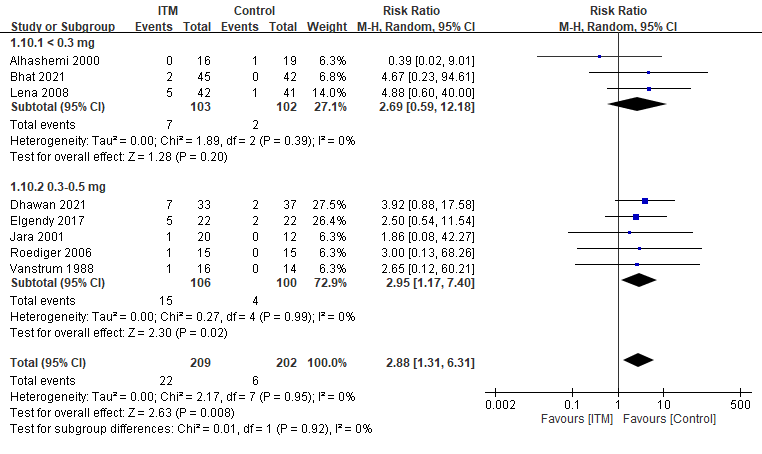


**Supplemental Figure 8**: Funnel plot for pain score at postoperative 12 hours.


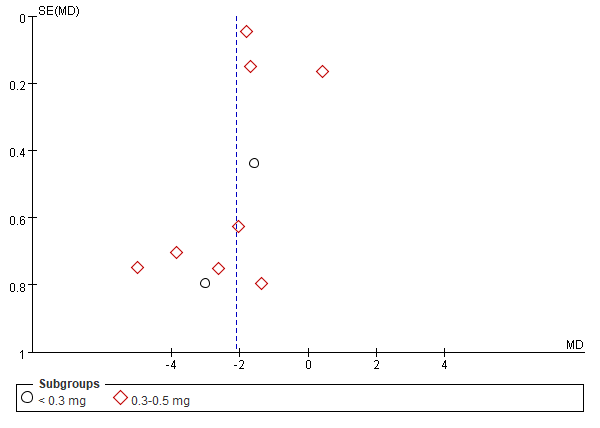


**Supplemental Figure 9**: Funnel plot for pain score at postoperative 24 hours.


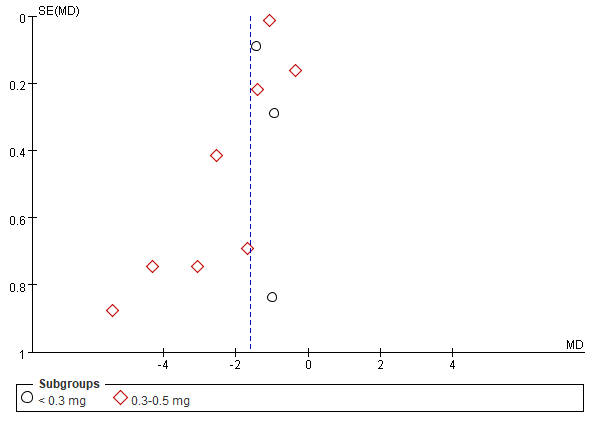


**Supplemental Figure 10**: Funnel plot for extubation time.


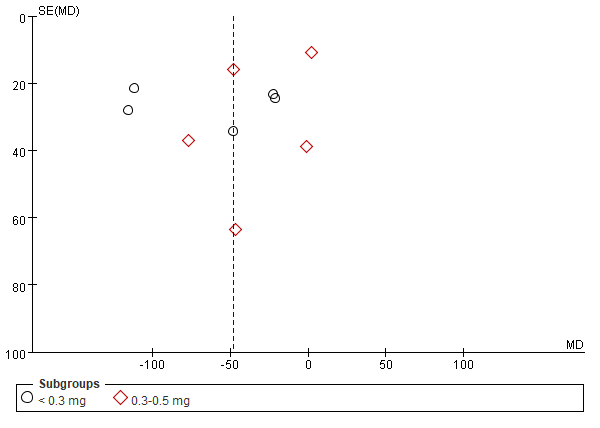


**Supplemental Figure 11**: Trial sequence analysis for pain score at postoperative 12 hours


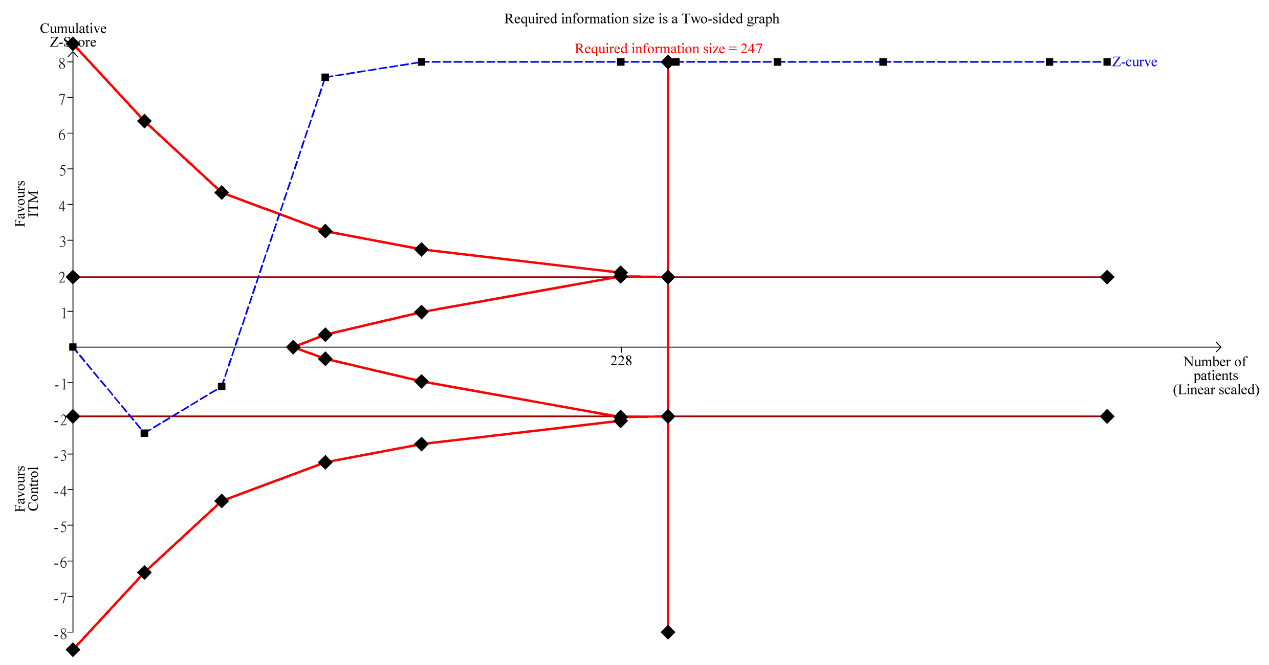


**Supplemental Figure 12**: Trial sequence analysis for pain score at postoperative 48 hours


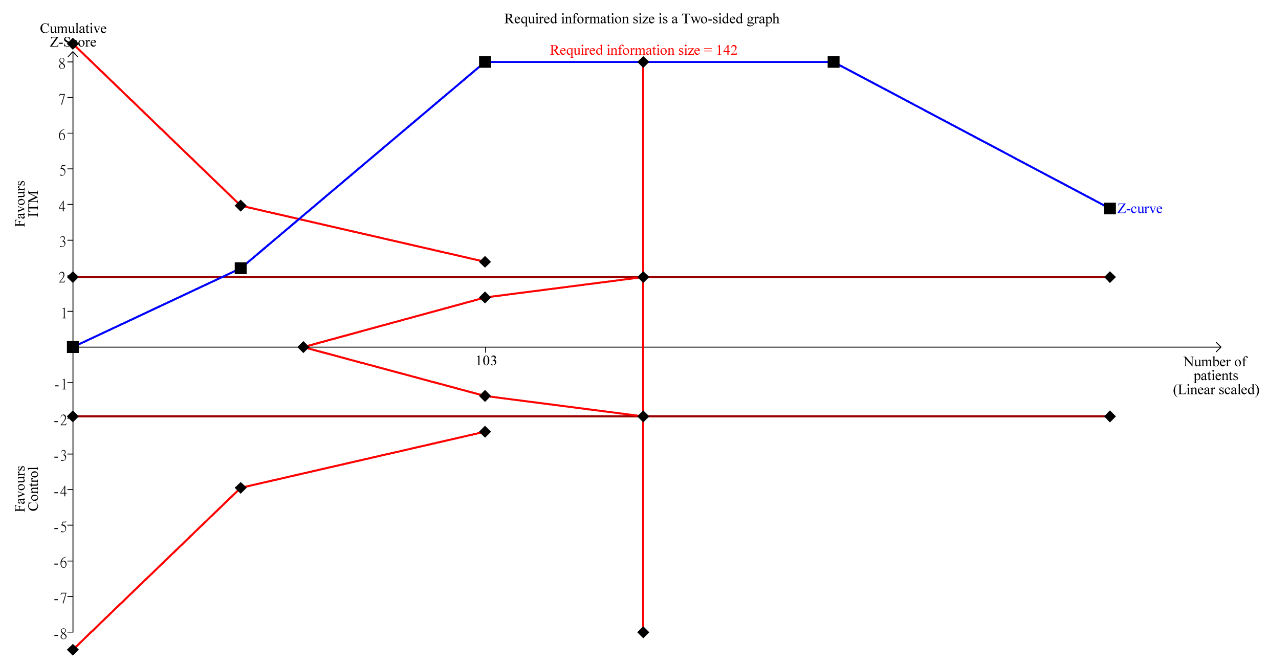


**Supplemental Figure 13.** Trial sequence analysis for morphine consumption at postoperative 24 hours


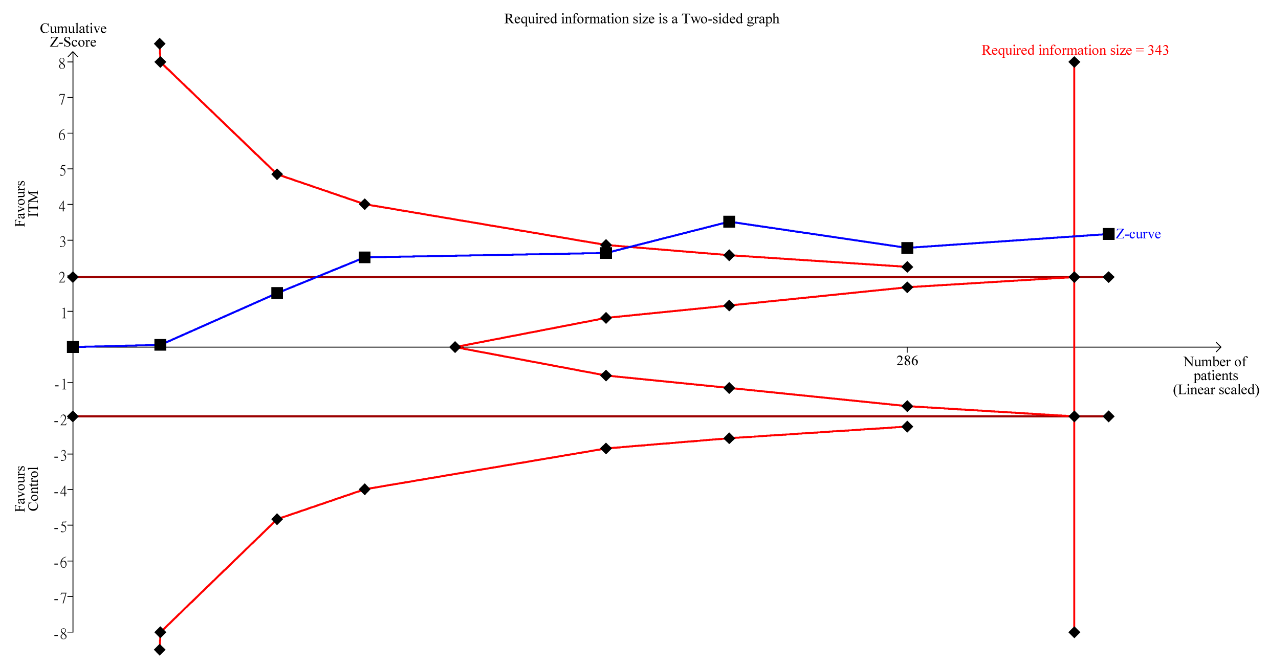


**Supplemental Figure 14.** Trial sequence analysis for morphine consumption at postoperative 48 hours


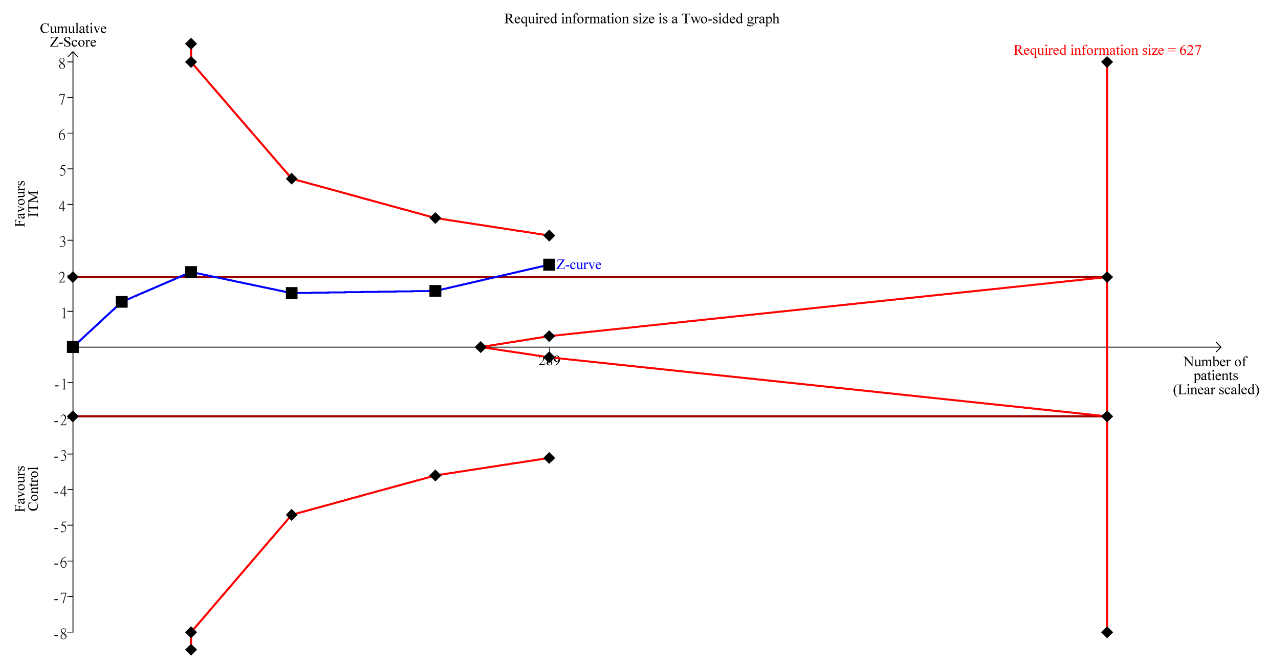


**Supplemental Figure 15.** Trial sequence analysis for extubation time


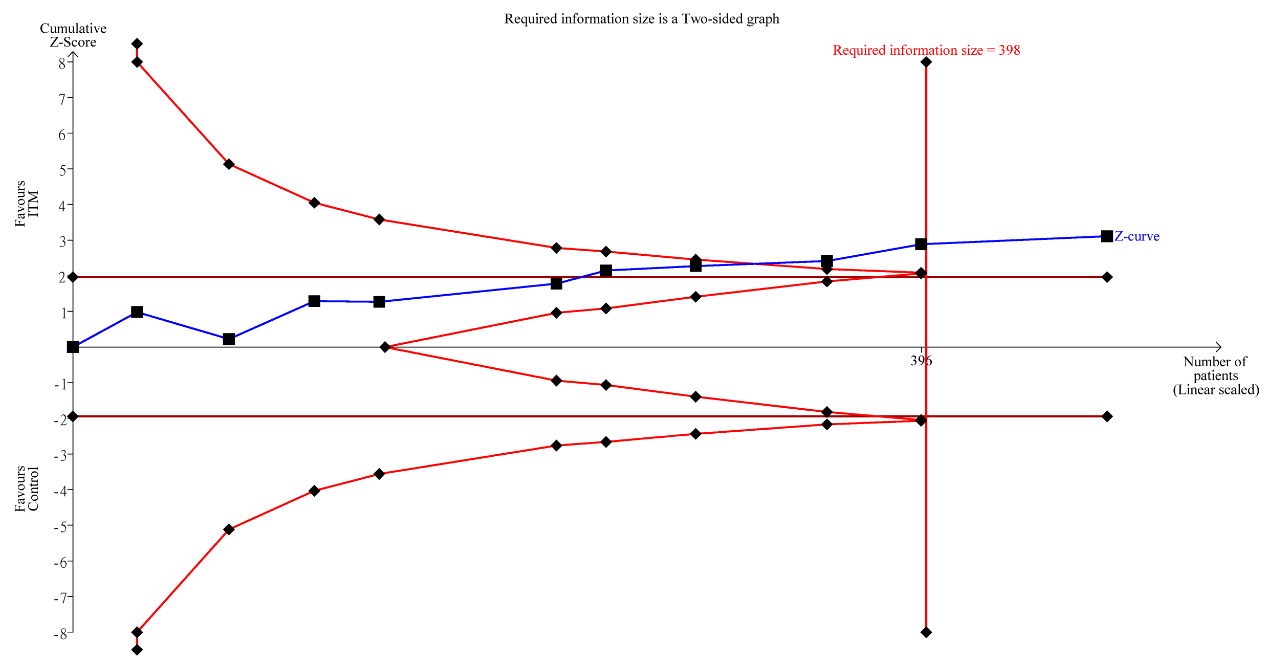


**Supplemental Figure 16.** Trial sequence analysis for intensive care unit stay


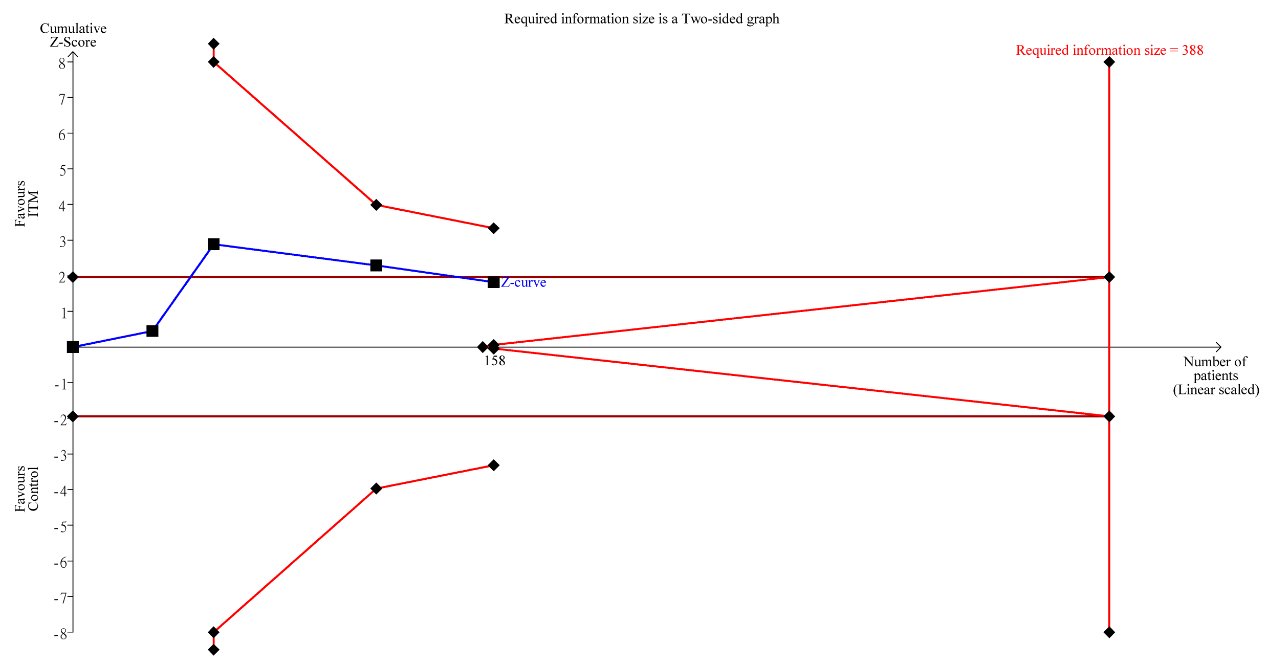


**Supplemental Figure 17.** Trial sequence analysis for hospital stay


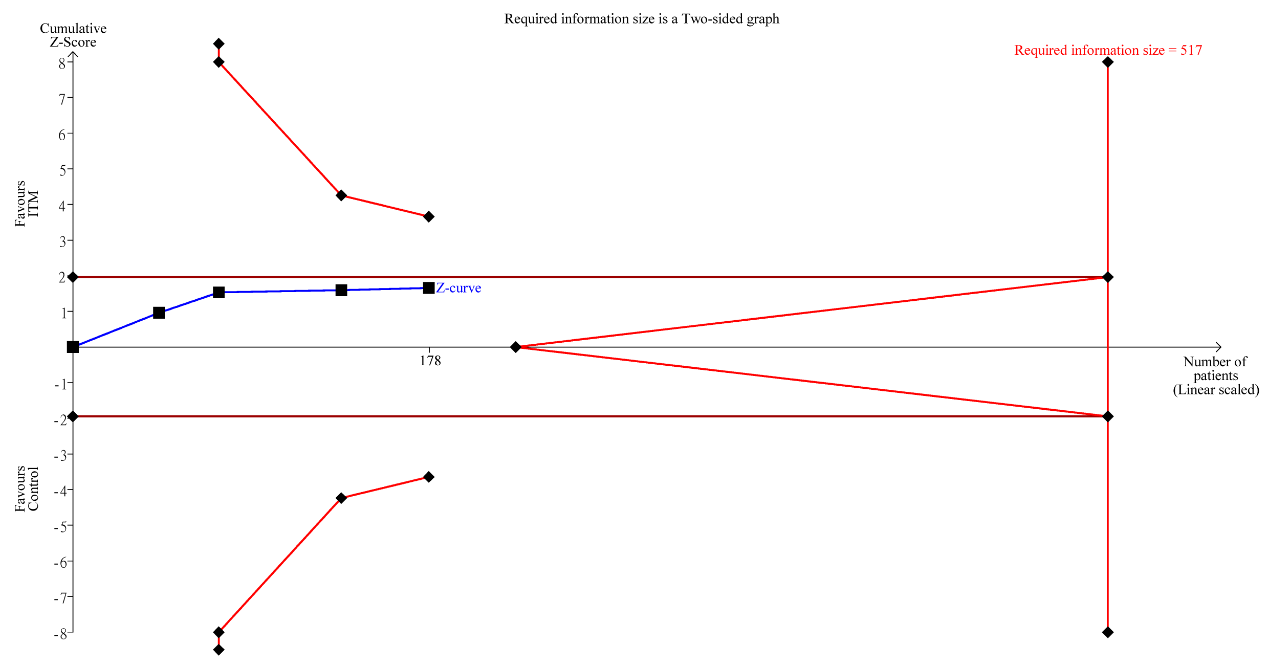


**Supplemental Figure 18.** Trial sequence analysis for postoperative nausea/vomiting


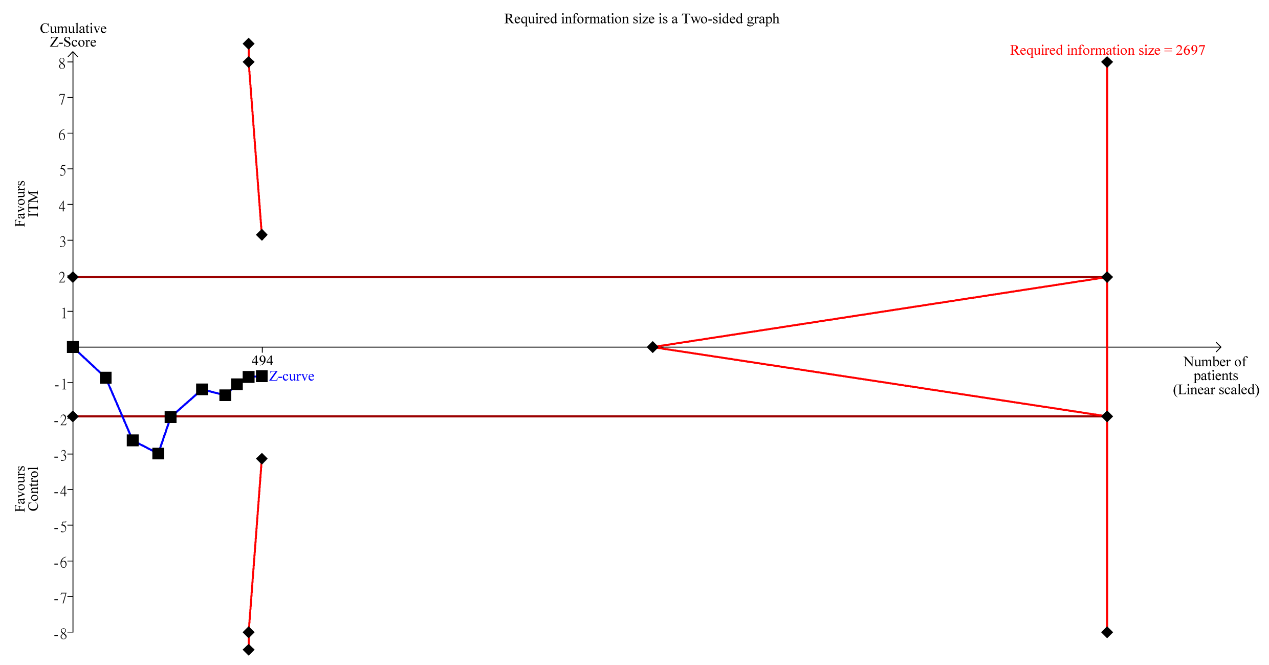


**Supplemental Figure 19.** Trial sequence analysis for postoperative pruritis


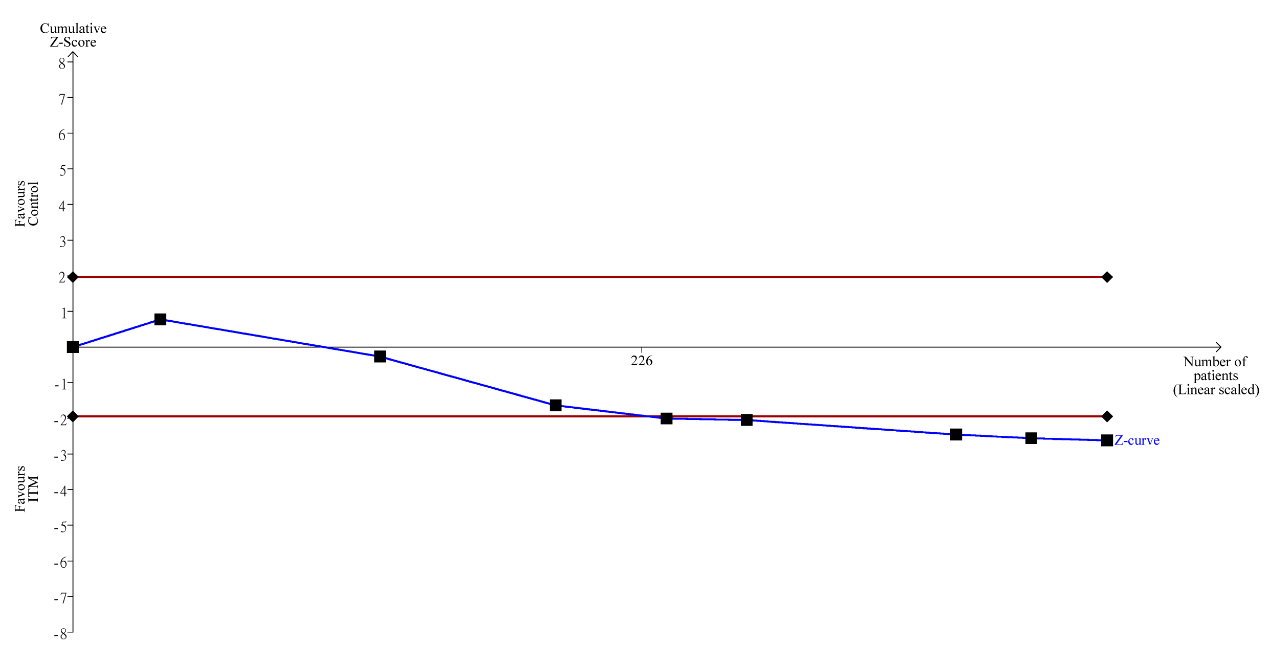

Supplement: Supplementary file 1 [file Data_Sheet_1.docx]
